# Exploring Senolytic and Senomorphic Properties of Medicinal Plants for Anti-Aging Therapies

**DOI:** 10.3390/ijms251910419

**Published:** 2024-09-27

**Authors:** Monika Imb, Zsolt Véghelyi, Michael Maurer, Harald Kühnel

**Affiliations:** Department of Applied Life Sciences, Bioengineering, University of Applied Sciences Campus Wien, Favoritenstraße 222, 1100 Vienna, Austria

**Keywords:** inflammation, herbal extracts, senolytics, senomorphics, chamomile, etoposide

## Abstract

Senolytic and senomorphic therapies have gained more and more attention in the last decade. This kind of therapy is based on the killing of cellular senescent cells without harming the “normal” cells. Aging is not a disease. Clinical studies on healthy people will be difficult to conduct. Therefore, one possibility is to draw on the large repertoire of medicinal plants and use their senolytic properties to provide mild anti-aging therapies. Chamomile, goldenrod, *reishi*, and green tea were tested for their ability to trigger senolysis. Quercetin was used as control substance. Cellular senescence was induced with 25 µM etoposide in human dermal fibroblasts and established for at least 14 days. The plant extracts were tested for their antioxidant potential (DPPH assay) and their polyphenol content. Senolysis was determined by presto blue assay of young and etoposide-induced senescent cells, and SA-β-Gal assays were also performed. The senomorphic properties of the plants were investigated using IL-6 ELISA and qPCR. It turned out that chamomile triggers a kind of cytokine storm and causes the cytokine values in the ELISA and in the qPCR to rise extremely, and other senescence-associated phenotype (SASP) markers were also elevated. Goldenrod and quercetin tend to have a senolytic and senomorphic effect, respectively. Regarding the senolytic and senomorphic properties of herbs, we found that all tested herbs can have a senolytic effect, and a senomorphic effect of quercetin has also been discovered. With regard to the effect of chamomile, however, we can say that seemingly harmless tea products may have harmful effects, especially in combination with chemotherapy, at least in cell culture experiments. Nevertheless, inflammation is a double-bladed mechanism with positive effects, for example, in healing, but also known negative effects.

## 1. Introduction

Cellular senescence is a state in the fate of cells first discovered by Hayflick and Morehead [1,2]. Originally, cellular senescence was described as an irreversible growth arrest, which appears to serve as a mechanism to prevent cancer. Cells enter this state when exposed to oncogenic stimuli, telomere shortening, or (sublethal) stress [3]. This growth arrest is primarily induced by two tumor suppressor pathways, p53/p21 and p16INK4a/pRB. Additionally, there are extensive changes in chromatin organization and gene expression. Furthermore, these cells exhibit a distinct phenotype characterized by enlarged and flattened morphology and increased senescence-associated β-galactosidase (SA-β-Gal) activity [4].

One notable change is the senescence-associated secretory phenotype (SASP), characterized by the secretion of numerous proinflammatory cytokines, chemokines, growth factors, and proteases. The SASP is not only involved in the development of cancer but also in its prevention [5]. Additionally, the SASP contributes to a systemic inflammatory state of aged humans and aging-associated diseases.

Cellular senescence can also be seen as a stress response involving at least three interacting signaling pathways: firstly, ongoing DNA damage response (DDR), which can be triggered by shortened telomeres, for example; secondly, senescence-associated mitochondrial dysfunction (SAMD), activated by the DDR and characterized by reduced respiratory activity, decreased membrane potential, and increased mitochondrial ROS production; and finally, the senescence-associated secretory phenotype (SASP), as mentioned previously [6].

Senescent cells persist within the organism and maintain the potential to modulate the tissue microenvironment [7]. Additionally, senescent cells exhibit increased resistance to apoptotic stimuli compared to their non-senescent counterparts [8]. This enhanced survival capacity of senescent cells is mediated by the upregulation of anti-apoptotic pathways, which allows them to persist within tissues [9].

There is strong evidence that senescent cell burden correlates with aging-associated diseases [10,11]. In response to the endurance of senescent cells and their potential detrimental effects, a new pharmacological strategy has emerged, known as senolytics. Senolytics are agents that selectively eliminate senescent cells by targeting the anti-apoptotic pathways, which confer resistance to cell death [12]. By selectively clearing senescent cells, senolytics aim to mitigate the adverse consequences associated with the accumulation of these cells during aging and in age-related diseases [13].

The development of senolytic drugs represents a promising approach for the treatment of various age-related pathologies [14], as the selective elimination of senescent cells has been shown to alleviate disease symptoms and improve tissue function in preclinical models [15]. By targeting the underlying cellular mechanisms that contribute to the persistence of senescent cells, senolytics offer a novel therapeutic strategy to address the complex challenges posed by age-related diseases [16].

A second approach is the prevention of the negative effects of the senescent cells. Therefore, medicines that target the SASP are one approach to alleviate the detrimental influence of senescent cells. These medicines are called senomorphics. Senomorphics are a class of agents that can modulate the phenotypes of senescent cells to resemble those of young cells without inducing apoptosis [17]. Unlike senolytics, which selectively eliminate senescent cells, senomorphics aim to suppress the harmful effects of the senescence-associated secretory phenotype (SASP) by interfering with senescence-related signaling pathways [18].

Senomorphics target various signaling cascades involved in cellular senescence, such as: mitogen-activated protein kinases (MAPKs), nuclear factor-kappa B (NF-κB), mammalian target of rapamycin (mTOR) and interleukin 1α (IL-1α). By modulating these pathways, as already mentioned, senomorphics can suppress the pro-inflammatory SASP without causing senescent cell death [19]. This approach may be particularly useful in situations where complete senescent cell clearance is undesirable or impractical.

Researchers are developing new clinical trial paradigms to evaluate the efficacy of senolytics and other interventions targeting fundamental aging processes. Traditional trials relying on long-term endpoints like lifespan or health span are often infeasible due to extended timelines and logistical challenges. Unfortunately, elderly subjects are generally excluded from clinical trials [20].

Examining the impact on the development and progression of multiple age-related diseases can provide insights into the broader effects on overall health and resilience. Accelerated aging models like progeria syndromes enable the study of rapid manifestation of age-related phenotypes to potentially slow or reverse underlying aging mechanisms. Focusing on diseases characterized by localized senescent cell accumulation allows us to investigate the ability of senolytics to selectively eliminate senescent cells and mitigate their detrimental effects. Trials are exploring the use of these interventions in potentially fatal diseases closely linked to senescent cell accumulation, aiming to improve outcomes and extend lifespan in affected individuals. Measuring functional capacity, physical performance, and resilience can provide insights into the potential of these therapies to maintain quality of life and independence in older adults. These emerging paradigms represent a shift in evaluating interventions targeting fundamental aging mechanisms. By assessing a range of age-related outcomes and conditions, researchers can gain a more comprehensive understanding of the potential benefits and applications of these innovative therapies [20].

Apart from this, one further possibility is searching for senolytic activity within medicines that have been used by humans for a long time. An example might be the application of herbal medicines that might have senolytic and/or senomorphic effects and a positive effect on health span. Substances derived from herbs like quercetin or fisetin are intensively studied in this regard [21,22,23], but it also makes sense to use extracts of parts of plants or the whole plant. These herbal medicines have been evaluated for hundreds of years. There is a lot of experience regarding medical herbs and their dosage and application, and because of this long-term use, they are easier to apply in terms of regulatory requirements.

Some extracts do already have proven effects on aging organisms, such as green tea [24] and goldenrod [25]. Another often-consumed aqueous extract is chamomile tea, which has known anti-inflammatory effects [26,27,28,29,30,31] Also, *reishi* mushroom, the so-called mushroom of eternal life, is used for treating elderly people in traditional medicine [32]. Active molecules include glycoproteins, polysaccharides, triterpenoids, steroids, and alkaloids, and many others. Apart from this, the literature lacks systematic investigations regarding senolytic and senomorphic effects of these extracts. The objective of this study was to investigate common herbal treatments for their ability to slow aging or extend health span. At least on the skin or in the digestive tract, there can be sufficient concentrations of these extracts to induce senolytic or senomorphic effects. We used commonly available herbal preparations bought in a drugstore for our investigations. We aimed to test effects of everyday usage of extracts on primary cells (human dermal fibroblast cells), which are the most frequently used models for cellular aging. Of course, in vitro effects of cell culture experiments cannot be directly be translated to “real life”, but basic molecular biological mechanisms can be observed and described for further investigations.

## 2. Results

### 2.1. Definition of Reagents

#### 2.1.1. Herbal Extracts and Solvents

**Figure 1:**
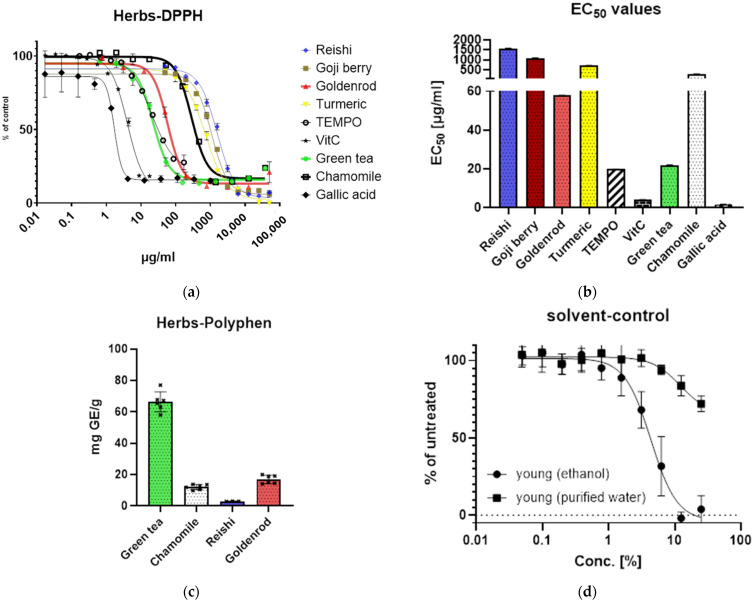
Definition of reagents and solvents: (**a**) DPPH assay of different herbs and antioxidants representing the antioxidative potential; % of controls labelling of x axis means that the values are expressed as percentage of abortion of untreated control (staining solution alone); (**b**) bar graph of EC_50_ of (**a**); (**c**) polyphenol content of extracts tested expressed in gallic acid equivalents (mg GE/g); mg gallic acid are determined by a standard curve with gallic acid standard compared to mg dry substance weighted in for preparation of extracts in the corresponding volume; (**d**) solvent control of purified water and ethanol (concentration expressed in % *v*/*v*) determined by XTT assay absorption values were related to the untreated control (% of untreated = value of treated/value of untreated × 100).

The antioxidative potential of different extracts was evaluated by DPPH assay and compared to the antioxidants ascorbic acid (vitamin C), TEMPO, and gallic acid. As expected, the *reishi* mushroom showed the least antioxidative potential, followed by the goji berry, and also turmeric is a little less antioxidative than chamomile. Goldenrod and even more green tea have an antioxidant potential comparable to TEMPO. Ascorbic acid and gallic acid show a response at very low concentrations (Figure 1a).

For EC_50_ values, *reishi* was 1569 µg/mL, goji berry 1083 µg/mL, goldenrod 57.89 µg/mL, turmeric 714 µg/mL, Tempo 19.82 µg/mL, vitamin C 4.039 µg/mL, green tea 21.87 µg/mL, chamomile 285.2 µg/mL, and gallic acid 1.688 µg/mL (see Figure 1b).

Polyphenol content was assayed by the Folin–Ciocâlteu method. Green tea has a polyphenol content of 66.50 ± 6.308 mg gallic acid equivalent (GE)/g (herb or mushroom used for preparing the extract) and chamomile 12.09 ± 1.615 mg GE/g. Furthermore *reishi* has the least content, with about 2.947 ± 0.08622 mg GE/g, and goldenrod has 16.97 ± 2.638 mg GE/g (Figure 1c).

Additionally, the optimal solvent concentration was determined (Figure 1d) by serial dilutions of purified water and ethanol (100%) in culture medium by XTT assay. We determined the maximum concentration of solvent added to the cells in our experiments. These concentrations were for 1.5% ethanol and 10% for water.

#### 2.1.2. Cell Lines and Establishment of the Senescent State

**Figure 2:**
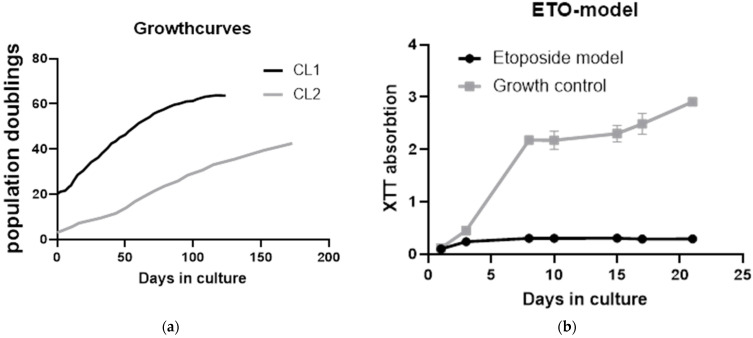
Growth curves. (**a**) Serial passaging of CL1 and CL2 (human dermal fibroblast cells from different donors and companies). Black line shows cell line with faster growth, which reached replicative senescence, gray line shows CL2, which had a lower growth rate and did not reach cellular senescence within the period we passaged the cells. (**b**) Confirmation of growth arrest induced by etoposide treatment by XTT assay. Ongoing growth of untreated cells can be seen (gray line with boxes), and the black line with circles shows growth arrest of etoposide-treated cells.

Growth curves of cell lines were established for CL1 (EverCyte HDF164 Vienna, Austria) and CL2 (ATCC), published in Kühnel et al. [33]. All experiments were performed at least 50% before the end of their replicative lifespan (replicative senescence (RS)). CL1 grew faster than CL2, which had quite a low doubling rate. Both cell lines were passaged for about 40 population doublings. CL2 did not reach RS in the time that it was cultivated (Figure 2a).

Etoposide-induced senescence (ES) was investigated with an XTT assay to show growth arrest. While growth control continued to increase to an absorption of above 2 and reached confluence, the etoposide (25 µM)-treated HDF cell line stopped growing after treatment for 48 h (Figure 2b). Data presented are from CL1, but growth curves and XTT assays were also performed with CL2 with the same results [33]. Detailed evaluation of the etoposide premature senescence model can also be found in our previous work [33].

### 2.2. Toxicology of Herbal Extracts

**Figure 3:**
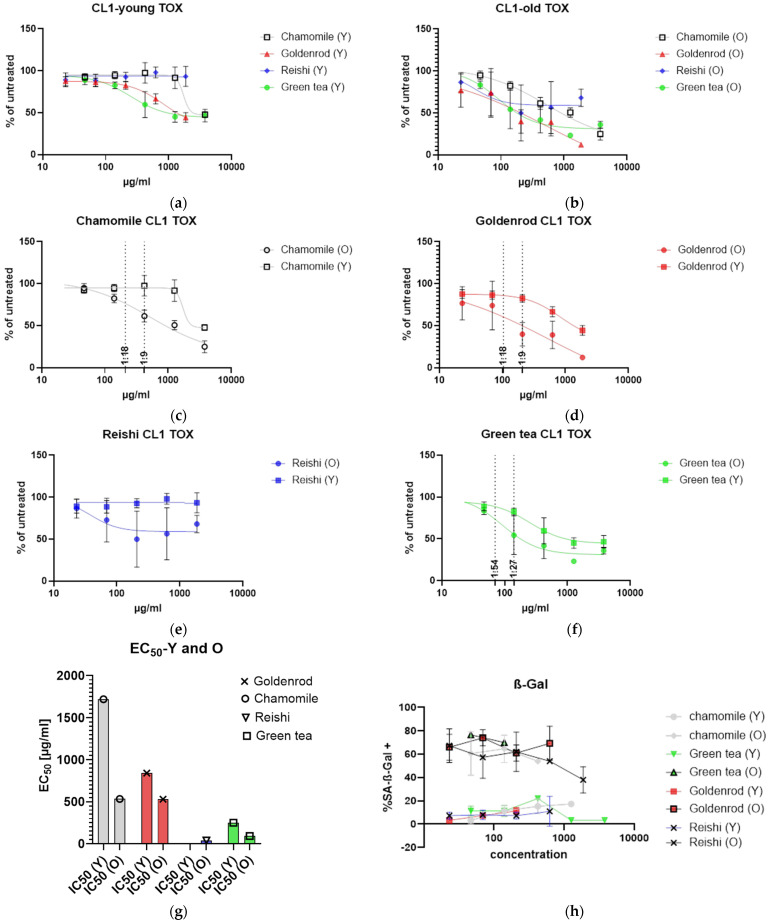
Toxicity of extracts: (**a**) young cells of CL1 treated with extracts of chamomile, goldenrod, *reishi*, and green tea; (**b**) old cells (etoposide-treated) with confirmed establishment of cellular senescence treated with the same extracts. (**c**–**f**) Single graphs of the extract treatments: (**c**) chamomile, (**d**) goldenrod, (**e**) *reishi*, and (**f**) green tea; (**g**) summary of EC50 values for young (Y) and old (O) in a bar graph; EC50 values are lower in the old treatment group; (**h**) SA-β-Gal-positive cell count of treated young and old cells.

Toxicology was assayed by presto blue assay. Cells with low passage number (Y) were seeded in a 24-well format and six different concentrations were tested in duplicate in three independent experiments. Untreated control cells were set at 100% and the rest of the samples were related to that (% of untreated = value of treated/value of untreated × 100). Young cells were affected by herbal extracts, but *reishi* showed no effect (Figure 3a). Figure 3b shows the same experiment with etoposide-induced senescent cells. Cells were treated with extracts 12 days after the end of etoposide treatment (2 days), and extracts were left on cells for 48 h. A clear shift to the lower left can be seen, indicating higher toxicity at lower concentrations.

Looking at each herbal extract separately, Figure 3c–f show a slight senolytic effect of all extracts. Concentrations for further treatment were determined by looking at the least nontoxic condition in young cells, marked by a dotted line at the corresponding concentration level.

Figure 3g presents the IC_50_ values in a column graph. The same treatment was also performed, with SA-β-Gal staining as final readout (Figure 3h). This analysis showed that young cells, as expected, had a lower percentage of SA-β-Gal-positive cells. At higher concentrations, some of the old cells died. For this reason, some of the lines (old and young cells) were not continued at higher concentrations. Interestingly, the mushroom extract showed a decreasing percentage of senescent cells. We will look at these effects in a further study, investigating the effects of *reishi* in more detail.

What can clearly be seen is that IC_50_ values decrease when old cells are treated with herbal extracts and mushroom extract, and there is a clear window opening up (EC_50_ (young cells) − EC_50_ (etoposide treated old cells)) between young and old cells. The largest window is formed by chamomile treatment (1185.2 µg/mL), but goldenrod also shows a senolytic effect, with a 311.1 µg/mL difference between young and old cells. Green tea shows a difference between old and young cells of 158.13 µg/mL, which is the lowest senolytic effect (Table 1).

### 2.3. Chronic Extract Treatment Cell Line 1

#### 2.3.1. IL-6 Secretion (SASP Formation)

IL-6, the most prominent and dominant protein of the SASP for human dermal fibroblasts according to our own data [33], was tested by ELISA. IL-6 values are expressed in ng/mL.

**Figure 4:**
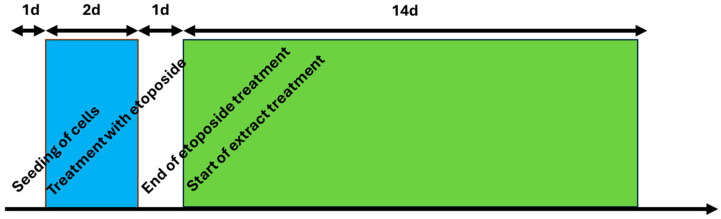
Chronic treatment of cells. Cells were seeded on day 0 and attachment took one day, then cells were treated with etoposide for 2 days (48 h) represented by the blue box. After a gap of 1 day, herbal treatment was started and proceeded for 14 days (green box), with media changed and fresh extracts added every 3–5 days.

**Figure 5:**
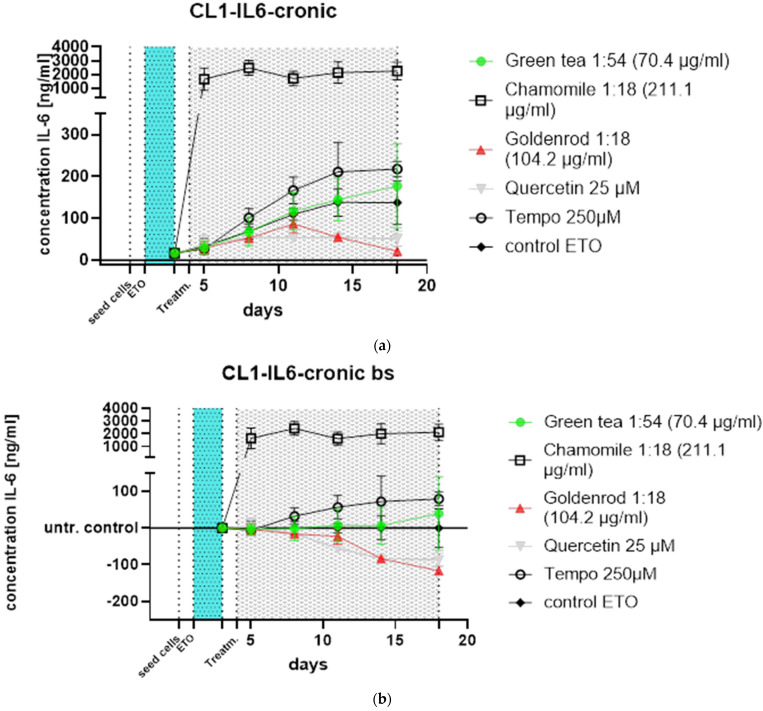
Time course of chronic treatments of HDF CL1. In both graphs, seeding of cells is marked by a dotted line at time point zero, and etoposide treatment is represented by the light-blue area from day 1 to 3 also between doted lines at the beginning and the end of the area. After one day of regeneration, the treatment was started, also indicated by a gray area from day 4 to 18 delimited by dotted lines indicating start and endpoint of the treatment, which was the end of this experiment. (**a**) Values detected by ELISA. Chamomile shows a massive increase in IL-6 excretion, and quercetin and goldenrod show decreased IL-6 production for different reasons explained in the text. (**b**) When normalized to ETO control, alleviating and worsening effects of extracts can be seen even better.

The course of treatment can be seen in Figure 4. The following treatments in Figure 5a were carried out: green tea in a concentration of 70.4 µg green tea/mL, which corresponds to a dilution of the original extract of 1:54; 211.1 µg chamomile flowers/mL, which corresponds to a dilution of 1:18; and goldenrod with a concentration of 104.2 µg/mL (also 1:18). A 25 µM quercetin solution was tested as a positive control and pure substance, and Tempo with 250 µM was tested as a control for antioxidant effects. As a further control, cells were treated only with etoposide and received no extract or substance treatment. Instead of herbal extracts only, the vehicle (water at a concentration of 10%) was added to this control. 

To make the effects more visible (Figure 5b), the treatments were related to the control not treated with extracts or pure substances at each time point. The effects are more visible here. It can clearly be seen that the chamomile (squares without filling) triggers a massive IL-6 reaction. A slight increase in IL-6 is also caused by the antioxidant Tempo (circles without filling). This illustration shows that the green tea (green dots) hardly reacts differently than if no extract is used: only the value after 18 days is increased. The effects of goldenrod and quercetin have already been described in detail in the abstract but are also better recognizable in this illustration. 

**Figure 6:**
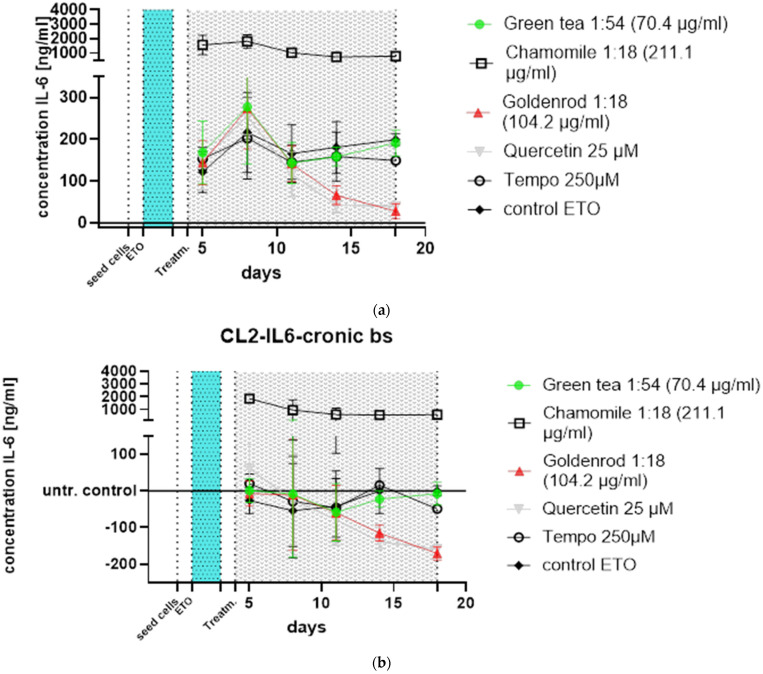
Time course of chronic treatments of HDF CL2. (**a**) Values detected by ELISA. Chamomile shows a massive increase in IL-6 excretion, and quercetin and goldenrod also show decreased IL-6 production in CL2 because of senomorphic and senolytic properties, respectively. (**b**) Data normalized to control (blank subtracted (bs)-control ETO) treatment illustrate the alleviating effects of quercetin and the massive induction of SASP by chamomile.

All experiments were confirmed with a second cell line (CL2), and the data can be found in Figure 6a,b. The pattern of the time course looks a little different, but the main outcome is the same: chamomile is massively inducing IL-6 (SASP). Quercetin has a senomorphic effect on the CL2 as well.

**Figure 7:**
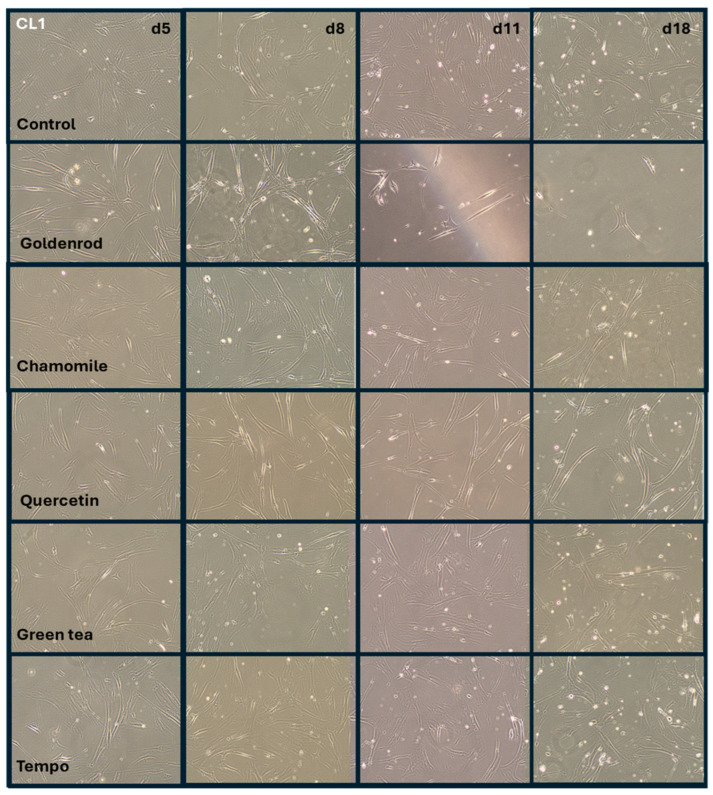
Images of the cells from the experiment described in Section 2.3.1 (IL-6 secretion (SASP formation)). Rows show different treatments and columns on the days when pictures were taken. Row one shows cells treated with etoposide, but that received no extract treatment. The following rows were treated with the substances or extracts indicated in the leftmost boxes.

**Figure 8:**
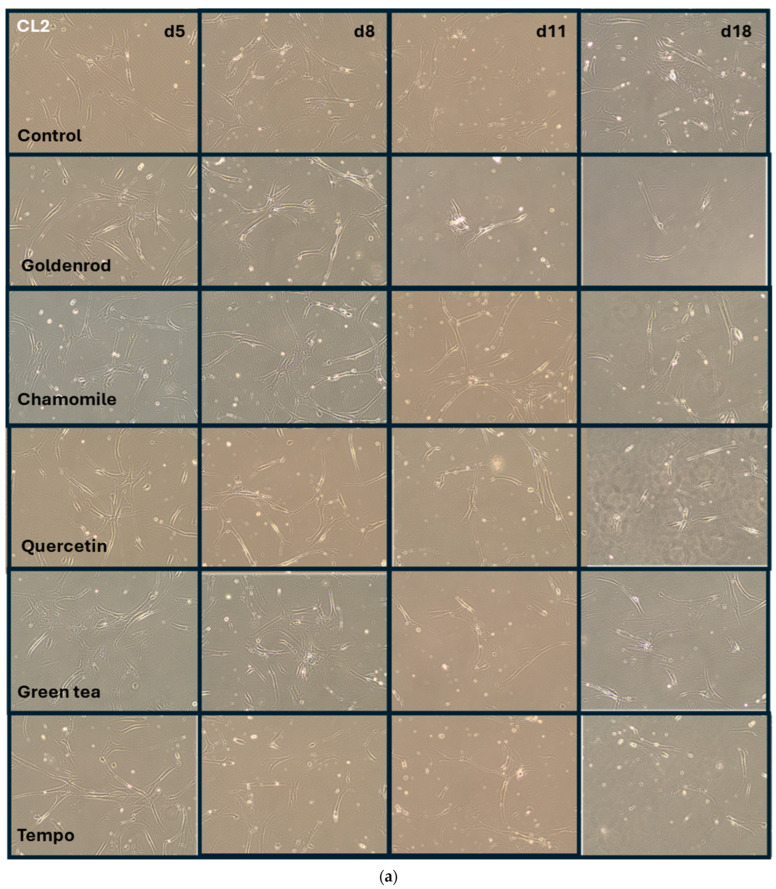

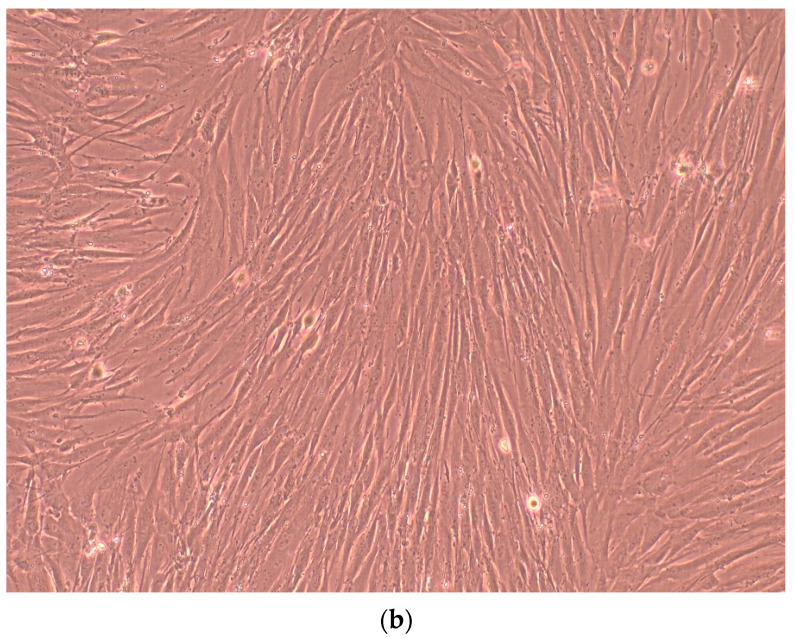
Images of the cells from experiment described in Section 2.3.1 (IL-6 secretion (SASP formation)). (**a**) Rows show different treatments and columns the days when pictures were taken. Cells have less seeding density than in Figure 7, but induction of senescence worked, as can be seen by arrested growth and senescent morphology (flattened appearance and less contrast of cells). Image of fully confluent fibroblast culture five days after seeding (**b**) Non etoposide treated cells reach confluence after this time period. The difference in confluence can be clearly seen compared to the even longer incubated cells after etoposide treatment.

Green tea (green dots) showed almost no difference from the control (black angled cubes) without plant extract. The chamomile (black cubes without filling), however, showed a massive increase in IL-6 concentration in the supernatants of the cell culture. The goldenrod (red triangles) showed a decrease in IL-6 concentration, but this seems to be due to a senolytic and not a senomorphic effect, as can be clearly seen from the reduced cell density on days 11 and 18 in Figure 7 and Figure 8. Quercetin (gray triangles) had a senomorphic effect in our setup and decreased IL-6 concentration compared to control without extracts.

#### 2.3.2. Cell Confluence and Morphology

Figure 7 shows the shape, density, and morphology of cells in representative images. Cell line 1 (control), which was treated with etoposide, but did not receive any herb or pure substance treatment, is in the uppermost row. Cells are arrested in growth and do not become dense in the time period of 18 days, while the confluence of cells stays nearly the same. In contrast, row two (cells treated with goldenrod) shows a clear decrease in density on days 11 and 18. This explains the reduction in IL-6 measured by ELISA, as mentioned in the preceding section. All the other treatments with chamomile, quercetin, green tea, and Tempo show the same morphology, shape, and confluence as the control cells. All experiments were confirmed with a second cell line (CL2), and the images can be found in Figure 8.

#### 2.3.3. qPCR Investigations of SASP-Related mRNA Expression

**Figure 9:**
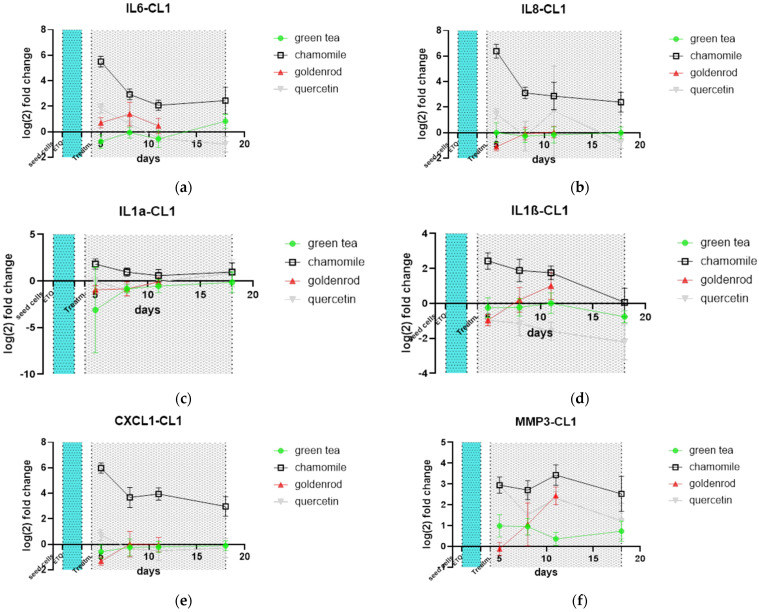
SASP factors determined by mRNA qPCR: (**a**) IL-6; (**b**) IL-8; (**c**) IL-1α; (**d**) IL-1β; (**e**) CXCL1; (**f**) MMP3. Values are normalized to control (not extract-treated). Day 18 of goldenrod was not included because of unreliable data and few cells. Start and end points of treatments are indicated by dotted lines. Etoposide treatment is indicated by a blue area as well the treatment with herbs is indicated by a gray area from day 4–18.

Important molecules of the SASP were investigated by qPCR on mRNA expression level. Levels of SASP factors were normalized to control (not extract-treated) to make effects more visible. IL-6 was upregulated at the mRNA level by chamomile treatment, as expected (Figure 9a). Furthermore, IL-8, IL-1α and β, and CXCL-1 also increased (Figure 9b–e). MMP3 as a metalloprotease representative was also increased by chamomile treatment (Figure 9f). For the other herbs and pure substances, there is no pronounced difference from control treatment, except for quercetin, which also showed an induction of expression of this metalloprotease. The quercetin-treated group showed decreased Il-1β expression at all time points. The late time points of goldenrod were not plotted because of limited cell numbers due to its senolytic effect, as can be seen in images of Figure 7 and Figure 8.

#### 2.3.4. Cell Line 1 Receptors

**Figure 10:**
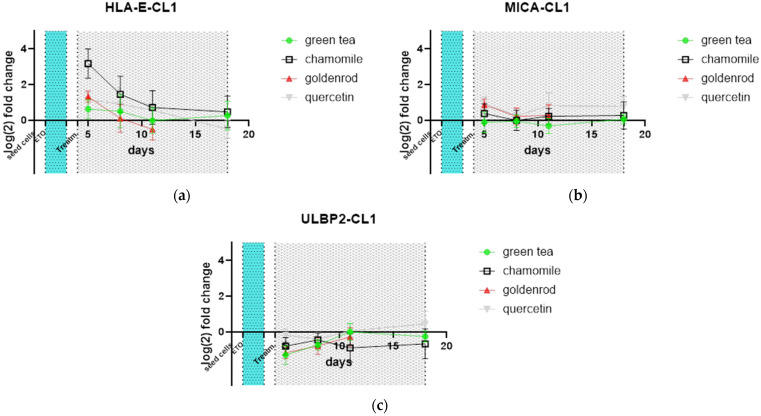
Receptor expression of mRNA analyzed by qPCR: (**a**) HLA-E; (**b**) MICA; (**c**) ULBP2.

Relevant receptors for immune surveillance and NK-cell degradation were also tested by qPCR. HLA-E (Figure 10a) was upregulated by chamomile treatment, while the other extracts did not show any pronounced reaction. No treatment altered the expression of MICA (Figure 10b) or ULBP2 (Figure 10c) compared to the non-extract-treated control. All values were again normalized to the non-extract-treated control.

#### 2.3.5. Senescence-Related Genes (CL1)

**Figure 11:**
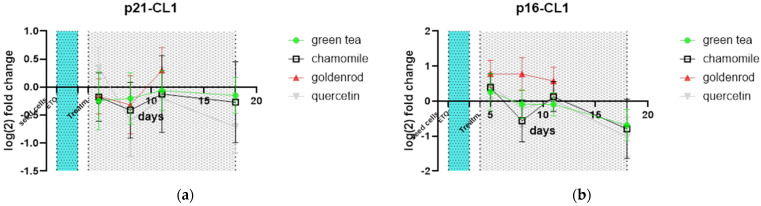
Senescence-related genes’ (**a**) p21 and (**b**) p16 values normalized to non-extract-treated control.

There was nearly no difference from the non-extract-treated control in p21 (Figure 11a) or p16 (Figure 11b). All treatments showed comparable expression of cell cycle-related genes.

### 2.4. Early and Late Treatment with Herbal Extracts

**Figure 12:**
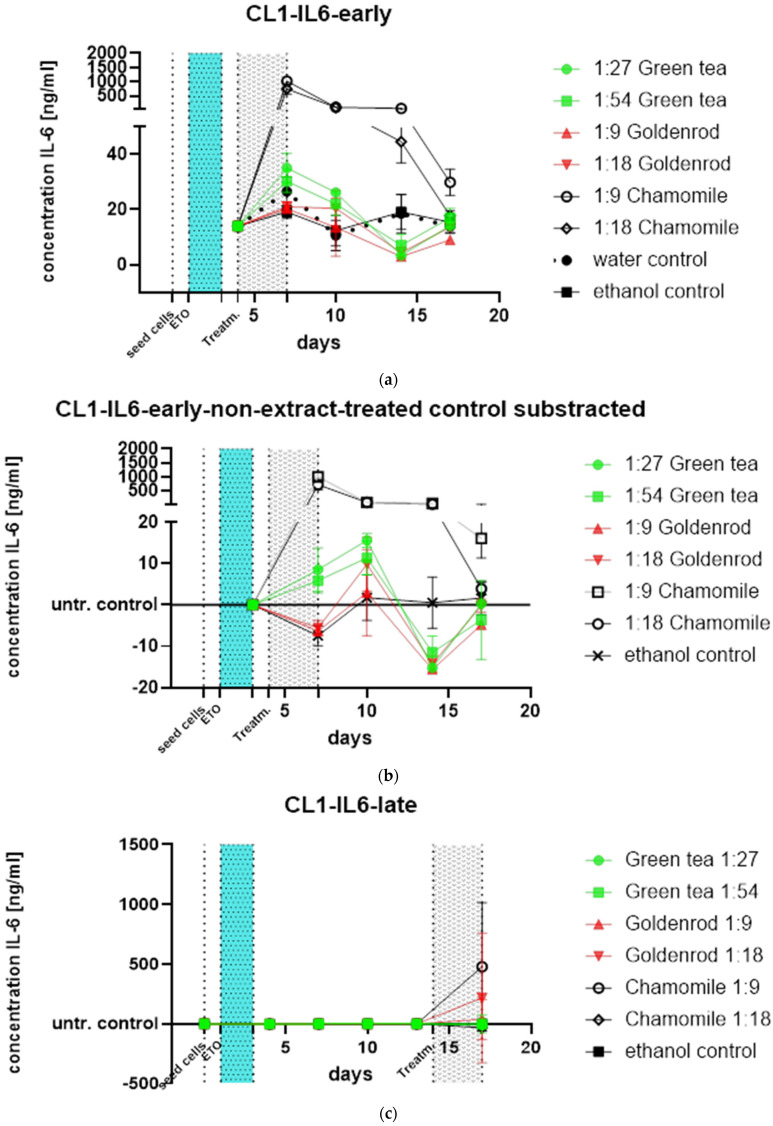
Treatments, late and early, showing short time exposure effects on damaged and etoposide-induced senescent cells; (**a**) early treatment was performed after 2 days of etoposide treatment 25 µM (light-blue area) and 1 day of regeneration for 3 days (gray area); (**b**) water control (non-extract treatment)-subtracted data, showing the differential expression of IL-6 protein compared to control; (**c**) late treatment (control-subtracted) indicates treatment of cells that are already etoposide-induced senescent. Here it can be seen that chamomile also induces an IL-6 response, but with a profound standard deviation (experiment was performed on just one biological replicate).

Additional treatments were performed. Non-chronic extract treatment was administered via an early pulse of extracts for three days after etoposide treatment (light-blue area in Figure 12a). Chamomile treatment upregulated IL-6 protein expression again: both dilutions (1:9 and 1:18) 211.1 µg/mL and 422.2 µg/mL increased the IL-6 value measured by ELISA above 1000 ng/mL. The IL-6 concentration of the 1:18 treatment returned to the level of the other treatments after 18 days, while that of the 1:8 treatment stayed a little bit higher. The other herbal treatments showed similar behavior to the water control (etoposide, but non-extract-treated). Here, we included an ethanol control where the cells were treated with the same ethanol concentrations, as in the goldenrod treatment. For better visualization of effects, we subtracted the non-extract-treated control (etoposide-treated, but instead of extracts, water-treated), as can be seen in Figure 12b. All extracts except chamomile were in the region of the control treatment and did not show notable senomorphic effects from this type of treatment.

The late treatment in Figure 12c, which shows the reaction of already senescent cells, was performed only once in triplicate. Chamomile also showed increased values of IL-6 with this kind of treatment, but with a huge standard deviation.

## 3. Discussion

This study aimed to show senolytic and/or senomorphic effects of plant and fungal extracts. A senolytic effect was shown for all tested extracts. For the senomorphic effect, an unexpected effect was shown. Plant and fungal extracts are described in the literature almost exclusively as anti-inflammatory and antioxidative. Almost nothing can be found about pro-inflammatory effects, but we saw that chamomile induced a massive inflammatory effect in etoposide-treated dermal fibroblast cells. Anti-inflammatory effects were observed in quercetin-treated cells.

For defining properties of extracts, the antioxidant potential was measured using the DPPH assay. Antioxidants can have an impact on cellular senescence by scavenging of reactive oxygen species of mitochondrial origin, thereby reducing senescence-associated mitochondrial dysfunction (SAMD) and interrupting the feedback loop between DNA damage and progressive senescence, thus alleviating SASP effects.

Antioxidants such as enzymes, mitochondria-targeting compounds, vitamins, carotenoids, organosulfur compounds, nitrogen-containing non-protein molecules, minerals, flavonoids, and non-flavonoids have been studied for their potential to counteract oxidative stress-induced senescence and modulate the SASP [34].

We used the substance Tempo as a control for the effect of antioxidants. Here, however, we were unable to observe any significant effects due to the purely antioxidant effect. This suggests that the observed effects follow mechanisms other than the elimination of radicals. Green tea had nearly the same antioxidative potential as Tempo (a stabilized radical that acts as radical scavenger and antioxidant) and thus the strongest antioxidant effect of all extracts, but hardly any effect in mitigating the SASP similarly to Tempo treatment. Tempo even had a small inducing effect in cell line 1 on IL-6 production in the long-term study, but the green tea showed no deviation from the non-extract-treated control in either cell line.

Polyphenols are important substances in the fight against cellular senescence. Polyphenol gallic acid also has the strongest antioxidative effect of the antioxidants we tested. For this reason, we measured the polyphenol content of our extracts. As expected, the Folin–Ciocâlteu assay showed hardly any reaction in the mushroom extract. In green tea, there are a lot of polyphenols present, but no direct correlation could be found between polyphenol content and senolytic or senomorphic effect either. Herbal preparations with high polyphenol content can also harm normal cells [35], especially in organs that are exposed to higher concentrations of extracts like the skin or the digestive system. Green tea also shows great toxicity in young and old cells (Figure 3f), which can be seen from the low IC_50_ values in Table 1. In this study, two primary human dermal fibroblast cell lines for checking the senolytic and senomorphic effects of extracts were used. HDF164 cells from Evercyte (CL1) were used in a patent (EP3643305A1) and the HDF cells from ATCC (CL2) were described in our former publication in detail [33]. Fibroblast cells of the skin are the most investigated model for cellular senescence and have been used in this regard since 1961 [1,2]. Because of the extremely long period of passaging the cells to induce replicative senescence, we were stressing the cells with chemotherapeutic etoposide to induce premature senescence. Etoposide is a topoisomerase II inhibitor and a known inducer of senescence [36] and the SASP [37]. Studies on replicative senescent cells and extract treatment would be very interesting, but were beyond the focus of this work. The effect of freshly damaged cells and the development of the SASP was the focus of this work. This research can also shed light on effects that might occur during and after chemotherapy [38]. Nevertheless, any senescence induction method might result in different kinds of SASP. The results of this study should be treated with caution due to the induction of senescence using chemotherapeutics. However, one advantage of the present study is that the development of senescence took place over a long period of time and the cells were given sufficient time to fully establish the state of cellular senescence.

For the interaction of etoposide with herbal extracts, it is known that herbal extracts can also increase the toxicity of chemotherapeutics like etoposide [39]. Some polyphenols found in herbal extracts can modulate etoposide-induced apoptosis. For example, resveratrol increases etoposide-induced apoptosis in certain cancer cell lines [40]. Some polyphenols like quercetin can inhibit etoposide-induced checkpoint kinase 1 (Chk1) phosphorylation [40], which is part of the DNA damage response. Also, etoposide induces cell death via mitochondrial-dependent actions. Some herbal extracts may interact with these pathways, potentially enhancing or modulating the effects of etoposide [41]. These effects may influence “young” cells differently from “old” cells, leading to the senolytic and senomorphic properties reflected in our toxicity studies. In this toxicity study, we compared low-passage cells to etoposide-induced senescent cells. All extracts we tested and showed some senolytic effect, which confirms the data of Lämmermann et al., who investigated the potential of goldenrod ethanolic extract [25]. Green tea and its ingredients are also known as a herbal extract with protective effect on the skin [42,43] and the aging brain [44], as well as having chemoprotective effects [45]. A study showed that doxorubicin-induced senescent fibroblasts developed an SASP and exhibited increased autophagy. Quercetin induced autophagy, increased ER stress, and partially triggered the death of senescent fibroblasts [46].

In this study, three different treatments were tested: the chronic treatment, which we focused most on, an early treatment, and a late treatment.

The chronic, long-term treatment was performed with two cell lines with three independent experiments for each. These time-consuming experiments were performed this way because we think that establishment of a senescent state needs some time, as realized in the study of Lämmermann et al. [25]. Often studies on senescent cells have been performed 1–3 days after exposure to damaging treatment [47,48]. We do not think that this is appropriate, especially since the SA-β-Gal assay often yields false positives when applied for a short time after damaging treatment (for example, with hydrogen peroxide) [49]. For this reason, we tried to prolong the time for establishment of senescence to at least 14 days. Unfortunately, we did not measure exact cell numbers in our chronic treatment experiment, but the images in Figure 7 and Figure 8 are representative and reflect the confluence and shape of the cells at the time points indicated. Furthermore, we defined the process of getting senescent after etoposide treatment well in our former study [33].

The effect of chamomile in the treatments performed is something that was not seen in former studies, though one study using treatments of sage (Salvia officinalis) extracts and chamomile (both 50 µg/mL) in combination with IL-1β treatment of human mature adipocytes found that after 4 h, IL-6 excretion had risen significantly compared to just IL-1β-treated cells. Sage even increased MCP-1, IL-6, IL-8 and ICAM-1 highly significantly in neuroblastoma (SK-N-SH) cells in a non-IL-1β treated group in 4 h and MCP-1 and ICAM-1 in 24 h. In combination with IL-1β treatment, mainly inflammatory markers decreased [50].

The massive upregulation of all tested SASP factors by chamomile following etoposide treatment is an effect that needs further investigation. The literature is full of antioxidative, anti-inflammatory, or antifibrotic properties of herbal extracts, but we could not find any hint to what we observed, neither with a conventional literature search nor with a KI-supported search (perplexity).

In the meantime, we have found a second herb with the same effects (*Sutherlandia frutescens* (L.)), but we have not been able to understand the mechanism of its effect yet. By already finding two herbs that induce this phenomenon, we concluded that it might be a not-so-uncommon effect. The effect of SASP factors secreted by fibroblast cells might have influences on cancer cells that grow nearby them.

Additionally, MICA was not induced by our treatment with chamomile (any of the other herbal treatments) compared to the non-herb-treated control. In the human organism, MICA binds to NKG2D, an important activating receptor on the surface of NK cells. Also, ULBP-2, which decreases susceptibility to natural killer cell-mediated cytotoxicity, is unaffected by chamomile treatment. These two receptors would lead to removal of senescent fibroblast cells [51]. Interestingly, the mediator of the opposing effect of HLA-E was induced in chamomile treatment. This would indicate that the senescent fibroblasts would remain in the tissue and could go on sending inflammatory signals to the surrounding tissue.

For quercetin, we found a clear senomorphic effect, as it clearly reduced IL-6 secretion in both primary cell lines. Quercetin can influence various cellular signaling pathways involved in senescence and cell survival and has antioxidant and anti-inflammatory effects by inhibiting inflammatory pathways. Quercetin may help mitigate the pro-inflammatory secretions of senescent cells.

We may have used excessive concentrations of goldenrod extract for investigations into senomorphic effects. Most of the cells died due to treatment with this extract. This confirmed senolytic activity, but could not shed light on any anti-inflammatory mode of action of this extract. The effects of goldenrod are investigated in greater detail elsewhere [25].

To sum up, we observed an effect of a herbal aqueous extract, which induced massive SASP protein production following a chemotherapeutic senescence induction. We do not know the mechanism yet, but we think it is worth further investigation. Regarding senolysis and senomorphic properties, we found that the herbs and mushroom tested can have a senolytic effect. A senomorphic effect of quercetin was also confirmed in our model. Regarding the effect of chamomile, we can say that seemingly harmless tea products can also have detrimental effects, perhaps especially in combination with chemotherapy, which must be confirmed in in vivo studies. Nevertheless, inflammation is a double-bladed mechanism, with positive effects, for example, in healing, but also known negative effects.

## 4. Materials and Methods

### 4.1. Cells

Primary cell line 1 (CL1), HDF164 human fibroblasts from dermal tissue at a population doubling level (PDL) of 20, were provided by Evercyte. Primary cell line 2 (CL2), human dermal fibroblasts at an initial PDL of 7, were obtained from ATCC. All the cells were cultured in growth medium (Dulbecco’s modified Eagle’s medium, prepared and sterile filtrated (0.2 µm)) containing 10% fetal calf serum (FCS), 100 U/mL streptomycin and 100 U/mL penicillin in a humified incubator at 37 °C and 5% CO_2_. The cells were grown until they reached 90% to 95% confluence and were passaged every 3 to 4 days. The total cell count and the viable cell count measurement was performed by a Spark^®^ Tecan (Männedorf, Seestrasse 103, Switzerland) microplate reader and trypan blue exclusion assay. Cell cultures were tested for mycoplasma using a MycoSPY^®^ (Biontex, München, Germany) PCR detection kit. Cells were passaged three times to obtain the cell bank for experiments. For all treatments, cells were seeded at 3500 cells/cm^2^ in all well plate and flask formats.

Etoposide was purchased from Sigma Aldrich (Art. E1383–25MG, Saint Louis, MO, USA). The stock solutions were prepared as follows: 25 mg etoposide was dissolved in 850 µL with a final concentration of 50 mM, aliquoted into 50 µL tubes, and stored at −20 °C. For experiments, etoposide was diluted 1:2000 to obtain a final treatment concentration of 25 µM. The cells were treated for 48 h to initiate the senescence process. After treatment, the etoposide-containing medium was removed and cells were washed with PBS.

#### 4.1.1. Toxicity Testing and SA-β-Gal Assay

Young cells were seeded in 24-well plates (3500 cells/cm^2^, 1000 µL medium per well). Aqueous herbal extracts (chamomile and green tea) were diluted 1:3 in water, and 100 µL of these dilutions was added to 900 µL medium in a 24-well plate. The goldenrod extract and the *reishi* extract were diluted 1:3 in 40% ethanol, and 37.5 µL of these extracts was added to 962.5 µL medium in a 24-well plate. The extract treatment for 48 h was started, beginning with 1:1, then 1:3, 1:9, 1:27, and 1:81 (i.e., 1:3 dilutions each). Row 6 contained blanks (purified water and EtOH 40%).

Presto blue staining was performed on day three: 500 µL old medium out, 500 µL fresh medium back in, 50 µL Presto Blue per well added, placed in the incubator for 30 min, then 100 µL each added to a black 96-well plate and fluorescence measured. Presto blue does not harm the cells, so the same 24-well plates were stained for SA-β-Gal the day after (day 4). Treatments represented the young treatment group.

The old treatment group was terminated as follows. Young cells were seeded in 24-well plates (3500 cells/cm^2^, 1000 µL medium per well). Next day, the cells were treated with etoposide for 48 h. After removal of etoposide, cells were incubated for 12 days with regular medium changing. Then, the extract treatment was started, with row 6 containing blanks (purified water and EtOH 40%). The first dilution step was 1:1, then 1:3, 1:9, 1:27, and 1:81 (i.e., 1:3 dilutions each). On day 14 after etoposide treatment, Presto blue staining was conducted, and the day after, SA-β-Gal staining.

#### 4.1.2. Main Experiment Chronic Extract Treatment for IL-6 ELISA and qPCR Investigations

Young cells were seeded in 75 cm^2^ flasks (3500 cells/cm^2^, 20 mL medium per flasks). One day after the seeding, the cells were treated with etoposide to induce senescence. After two days, etoposide was removed and fresh medium was added. The day after the removal of etoposide, the first extract treatment took place and supernatant was collected for the first time. From this point on, one flask per extract was harvested at each medium change/extract treatment, and both the RNA and supernatants were collected for further investigation. The process is explained in Figure 4.

### 4.2. Preparation of Extracts (Used Dilutions Can Be Found in Table 2)

Ethanol (EtOH) was the solvent of choice for all drugs except the green tea and the chamomile extracts, where purified water was used. To prepare the concentration of 40%, 300 mL of purified water was measured and mixed with 200 mL of ethanol absolute 99.9% p.A. to obtain 500 mL of 40% EtOH, and stored at room temperature in a brown bottle to prevent exposure to light as far as possible. To rule out any effect (positive or negative) of the solvents, cells were treated with solvents without extracts in each experimental setup.

Green tea from Kottas Pharma GmbH was purchased at a local pharmacy (Schubert Apotheke, Arndtstraße 88, 1120 Vienna, Austria). Two teabags (3.8 g) were steeped in 100 mL of purified water to simmer for five minutes and covered with aluminum foil to prevent any loss of liquid through evaporation. After steeping, the tea was filtered using a 0.45 µm syringe filter and sterile filtered afterwards. Aliquots were then frozen at −20 °C at a stock concentration of 38 g/L.

Dried chamomile flowers (*Matricaria chamomilla*) were purchased at a local pharmacy (Schubert Apotheke, Arndtstraße 88, 1120 Wien). Again, 3.8 g was weighed out and simmered gently with 100 mL of purified water for five minutes and covered with aluminum foil to prevent any loss of liquid through evaporation. After steeping, the extract was filtered using a 0.45 µm syringe filter and sterile filtered afterwards. Aliquots were then frozen at −20 °C at a stock concentration of 38 g/L.

Dried goldenrod herb (*Solidago virgaurea*) was purchased at a local pharmacy (Schubert Apotheke, Arndtstraße 88, 1120 Vienna, Austria), after which 2.5 g was weighed out, 50 mL of EtOH 40% added, and the combination ground in a mixer. This mixture was left to infuse overnight at 4 °C. After steeping, the extract was filtered using a 0.45 µm syringe filter and sterile filtered afterwards. Aliquots were then frozen at −20 °C at a stock concentration of 50 g/L.

Dried *reishi* (mushroom of eternal life) powder was provided by a drugstore (Schubert Apotheke, Arndtstraße 88, 1120 Vienna, Austria), after which 2.5 g was weighed out, 50 mL of EtOH 40% added, and the combination ground in a mixer for better dispersion of the fine powder. This mixture was left to infuse overnight at 4 °C. After steeping, the extract was filtered using a 0.45 µm syringe filter and sterile filtered afterwards. Aliquots were then frozen at −20 °C at a stock concentration of 50 g/L.

For dried curcumin powder (Schubert Apotheke, Arndtstraße 88, 1120 Vienna, Austria), 2.0 g was weighed out, 40 mL of EtOH 40% added, and the combination ground in a mixer for better dispersion of the fine powder. This mixture was left to infuse overnight at 4 °C. After steeping, the extract was filtered using a 0.45 µm syringe filter and sterile filtered afterwards. Aliquots were then frozen at −20 °C at a stock concentration of 50 g/L.

Dried goji berries (Lycium barbarum) (Schubert Apotheke, Arndtstraße 88, 1120 Vienna, Austria) were purchased at a drugstore, after which 2.5 g was weighed out, 50 mL of EtOH 40% added, and the combination ground in a mixer. This mixture was left to infuse overnight at 4 °C. After steeping, the extract was filtered using a 0.45 µm syringe filter and sterile filtered afterwards. Aliquots were then frozen at −20 °C at a stock concentration of 50 g/L.

**Table 2:** Dilutions and concentrations used for 24 well and 75 cm^2^ flask assays.

Chamomile	Green Tea	Goldenrod	*Reishi*
1:1 means 3.8 mg/mL	1:1 means 3.8 mg/mL	1:1 means 1.875 mg/mL	1:1 means 1.875 mg/mL
1:3 means 1.2666 mg/mL	1:3 means 1.2666 mg/mL	1:3 means 0.6250 mg/mL	1:3 means 0.6250 mg/mL
1:9 means 0.4222 mg/mL	1:9 means 0.4222 mg/mL	1:9 means 0.2083 mg/mL	1:9 means 0.2083 mg/mL
1:18 means 0.2111 mg/mL	1:27 means 0.1407 mg/mL	1:18 means 0.1042 mg/mL	1:27 means 0.0694 mg/mL
1:27 means 0.1407 mg/mL	1:54 means 0.0704 mg/mL	1:27 means 0.0694 mg/mL	1:81 means 0.0231 mg/mL
1:81 means 0.0469 mg/mL	1:81 means 0.0469 mg/mL	1:81 means 0.0231 mg/mL	

### 4.3. DPPH Assays

For the DPPH (2,2-diphenyl-1-picrylhydrazyl) assays, three different standard solutions were prepared: vitamin C (prepared from a 1000× stock solution in 40% EtOH), Tempo (prepared from a 100× stock solution in RO water), and gallic acid (prepared in RO water). DPPH was dissolved in methanol to obtain a stock solution with a concentration of 25 mM. Sample solutions (100 µL) were diluted with an equal volume of 0.25 mM stock solution, and 100 µL was mixed and incubated in the dark at room temperature for 30 min. The absorbance of the reaction mixture was measured at 517 nm using a photometer. The decrease in absorbance indicates the extent of the DPPH radical scavenging activity (all reagents from Sigma Aldrich, Saint Louis, MO, USA).

### 4.4. Folin–Ciocâlteu Assay

The Folin–Ciocâlteu assay was performed as follows. To measure the extract samples, a 1:3 dilution series was prepared. A standard curve was constructed using gallic acid. A volume of 25 µL of each extract in each dilution stage, 125 µL of Folin–Ciocâlteu phenol reagent, and 100 µL of sodium carbonate (20% Na_2_CO_3_ *w*/*v* in water) were prepared in a 2 mL tube, incubated for 5 min at 50 °C, and cooled in ice water afterwards for another 5 min, then 100 µL was transferred from the tubes to a 96-well microplate. Afterwards, the absorbance was measured at 760 nm. A calibration curve was constructed using standard solutions of gallic acid. The absorbance was plotted against the concentration of gallic acid to create a standard curve. The total phenolic content in the sample was determined by comparing the absorbance of the sample to the calibration curve. Results are expressed in milligrams gallic acid equivalent per gram of herbal substance. (all reagents from Sigma Aldrich, Saint Louis, MO, USA).

### 4.5. XTT (2,3-Bis-(2-Methoxy-4-Nitro-5-Sulfophenyl)-2H-Tetrazolium-5-Carboxanilide) Assay

The effect of solvent controls on cell viability and growth was evaluated three days after treatment by performing an XTT assay according to the manufacturer’s instructions. In brief, the XTT working solution was prepared mixing XTT labeling reagent and electron-coupling reagent at a ratio of 5:0.1 immediately before application. Of the 100 µL volume per well, 50 µL was discarded, and 50 µL XTT working solution was added to each well. Plates were incubated under standard cultivation conditions for 4 h. The absorption signal was measured at a wavelength of 450 nm using 650 nm as a reference. The absorption signal of the untreated control was set to 100% and the relative signal of solvent-treated replicates was calculated as a measure of viable cell numbers per well. (XTT reagent purchased from Sigma Aldrich Artikel # 11465015001 Roche).

### 4.6. Senescence-Associated β Galactosidase (SA-β-Gal) Assay

The cells were washed twice with PBS and fixed with a 4% formaldehyde fixation solution for 10 min at room temperature, washed again twice with PBS and once with staining buffer (citric acid 0,1 M phosphate buffer pH 6.0), and incubated with the X-Gal staining solution (5 mM K_4_[Fe(CN)_6_], 5 mM K_3_[Fe(CN)_6_], 2 mM MgCl_2_, 1 mg/mL X-Gal in staining buffer) for a minimum of six hours at 37 °C. The staining solution was removed, and the cells were then covered with 70% glycerol and stored at 4 °C for longer storage. To determine the percentage of β-Gal-positive cells, approximately 50 cells were counted per well in three to four different sections in the well’s center, since cells tend to be more likely to cluster in the center of a well than to migrate to the edge. This was carried out three times to minimize counting errors. (all reagents from Sigma Aldrich, Saint Louis, MO, USA).

### 4.7. IL-6 ELISA

For the measurement of IL-6 content in cell-conditioned media, an ELISA kit (Human IL-6 CytoSet™ from Invitrogen™, Carlsbad, CA, USA) was used. An anti-human IL-6 antibody (0.125 mg/0.125 mL) was used as the coating antibody. For detection, an anti-human IL-6 biotin antibody (0.25 mg/0.125 mL) was employed, with streptavidin–HRP facilitating detection. Recombinant human IL-6 was used for calibration. The IL-6 ELISA was carried out following the supplier’s instructions.

### 4.8. Presto Blue Assay

The PrestoBlue™ assay is a cell viability assay used to measure the metabolic activity of cells. It is based on the reduction of a resazurin-based reagent to a red fluorescent and colorimetric product, resorufin, by metabolically active cells. It enables the measurement of cell proliferation and serves as a reference for the cell count in each well. The intensity of the fluorescence or color is directly proportional to the number of viable cells. PrestoBlue™ cell viability reagent from Invitrogen™ was purchased. The dye was diluted 1:20 in medium, 1 mL (10% *v*/*v*) of this mixture was added to each well of the 24-well plates, and the plates were incubated at 37 °C for 30 min. The fluorescence was measured at 560 nm (purchased from Thermo scientific, Waltham, MA, USA).

### 4.9. RNA Extraction, cDNA Synthesis, and qPCR

Total RNA was extracted from HDF and HDFK cells using the Aurum^TM^ Total RNA mini kit (BIORAD, Hercules, CA, USA), following the instructions for cultured cell lines.

The cells were harvested, washed in PBS, and spun down in a microcentrifuge tube. The pellet from the cell culture flask was mixed with lysis buffer and 70% EtOH. This homogenized lysate was pipetted in an RNA-binding column standing in a capless wash tube and centrifuged for 30 s. After washing, the RNase-free DNase I and DNase dilution solutions were added and incubated for 15 min at room temperature to allow digestion. After two more washing steps, the RNA-binding column was transferred to a capped microcentrifuge tube and eluted after 1 min by centrifuging it for another 2 minutes. The tubes were then frozen at −20 °C for later use.

The cDNA synthesis was performed using an iScript^TM^ cDNA synthesis kit purchased from BIORAD, Hercules, CA, USA. The total RNA collected in Section 4.1.2 was reverse-transcribed with iScript reverse transcriptase and iScript reaction mix using the kit’s standard protocol and run in a thermal cycler: priming 5 min at 25 °C; reverse transcription 20 min at 46 °C; reverse-transcriptase inactivation 1 min at 95 °C. Per sample, 40 µL was frozen at −20 °C.

The cDNA samples from the previous step were diluted 1:3 with nuclease-free water. A primer mix consisting of forward and reverse primers in nuclease-free water with a desired final concentration of 400 nM was obtained. Each qPCR reaction consisted of a total volume of 10 μL containing 5 µL iTaq Universal SYBR Green super mix, 1 µL PCR primer mix, 2 μL cDNA template, and 2 µL of nuclease-free water. Housekeeping genes actin β and GAPDH were used. All samples were analyzed in duplicate. The PCR thermal cycle protocol consisted of the following: 1 cycle of 95 °C for 30 s, 40 cycles of 95 °C for 5 s, and 60 °C for 30 s. At the end of the run, a melting curve was assembled. Primer sequences and references can be found in Table 3.

### 4.10. Statistical Analysis

For x and y plots, average and standard deviation were calculated by GraphPad Prism 10 version 10.3.0 (507) 30 July 2024. For the toxicity testing, three samples in duplicates were analyzed, and for the qPCR analysis, two independent experiments with two technical replicates were performed. Data were calculated by the delta delta CT method with two different housekeeping genes (actin β and GAPDH). Nonlinear regression and IC_50_ calculations were also performed using this software three times in duplicate ((inhibitor) vs. response–variable slope (four parameters)).

## Figures and Tables

**Table 1:** IC_50_ values of tested substances.

	Chamomile	Goldenrod	*Reishi*	Green Tea
**IC_50_ (young cells)**	1719 µg/mL	841.6 µg/mL	Not stable	250.1 µg/mL
**IC_50_ (etoposide treated old cells)**	533.8 µg/mL	530.5 µg/mL	38.5 µg/mL	91.97 µg/mL

**Table 3:** Primer sequences and source.

Name	Forward (5′-3′)	Reverse (5′-3′)	Ref.
p16INK4a	GAGCAGCATGGAGCCTTC	CGTAACTATTCGGTGCGTTG	[52]
p21	GGCAGACCAGCATGACAGATTTC	CGGATTAGGGCTTCCTCTTGG	[53]
IL-1α	GGTTGAGTTTAAGCCAATCCA	TGCTGACCTAGGCTTGATGA	[52]
IL-1β	ACAGATGAAGTGCTCCTTCCA	GTCGGAGATTCGTAGCTGGAT	[54]
IL-6	GCCCAGCTATGAACTCCTTCT	GAAGGCAGCAGGCAACAC	[52]
IL-8	AGACAGCAGAGCACACAAGC	ATGGTTCCTTCCGGTGGT	[52]
CXCL1	GAAAGCTTGCCTCAATCCTG	CACCAGTGAGCTTCCTCCTC	[55]
GAPDH	CGACCACTTTGTCAAGCTCA	TGTGAGGAGGGGAGATTCAG	[56]
Actin β	CCAACCGCGAGAAGATGA	CCAGAGGCGTACAGGGATAG	[57]
MMP-3	CAAAACATATTTCTTTGTAGAGGACAA	TTCAGCTATTTGCTTGGGAAA	[52]
HLA-E	TGCGCGGCTACTACAATCAG	TGTCGCTCCACTCAGCCTTC	[58]
MICA	ATGGAACACAGCGGGAATCA	GCACTTTCCCAGAGGGCAC	[51]
ULBP2	TCCAGGCTCTCCTTCCATCA	AGAAGGATCTTGGTAGCGGC	[51]

## Data Availability

The original contributions presented in the study are included in the article, further inquiries can be directed to the corresponding author.
